# A method for temporal-spatial multivariate genomic analysis of acute wound healing via tissue stratification: a porcine negative pressure therapy pilot study

**DOI:** 10.3389/fmmed.2023.1195822

**Published:** 2023-08-31

**Authors:** Jacob G. Hodge, Sumedha Gunewardena, Richard A. Korentager, David S. Zamierowski, Jennifer L. Robinson, Adam J. Mellott

**Affiliations:** ^1^ Bioengineering Graduate Program, University of Kansas, Lawrence, KS, United States; ^2^ Department of Plastic Surgery, University of Kansas Medical Center, Kansas, KS, United States; ^3^ Department of Cell Biology and Physiology, University of Kansas Medical Center, Kansas, KS, United States; ^4^ Department of Chemical and Petroleum Engineering, University of Kansas, Lawrence, KS, United States; ^5^ Department of Orthopaedics and Sports Medicine, University of Washington, Seattle, WA, United States; ^6^ Department of Mechanical Engineering, University of Washington, Seattle, WA, United States; ^7^ Institute for Stem Cell and Regenerative Medicine, University of Washington, Seattle, WA, United States; ^8^ Ronawk Inc., Olathe, KS, United States

**Keywords:** genomics, negative pressure wound therapy, porcine model, wound healing, incisional wounds, bioinformatics

## Abstract

**Introduction:** Wound therapies are capable of modulating the complex molecular signaling profile of tissue regeneration. However traditional, bulk tissue analysis results in nonspecific expressional profiles and diluted signaling that lacks temporal-spatial information.

**Methods:** An acute incisional porcine wound model was developed in the context of negative pressure wound therapy (NPWT). Dressing materials were inserted into wounds with or without NPWT exposure and evaluated over 8-hours. Upon wound explantation, tissue was stratified and dissected into the epidermis, dermis, or subcutaneous layer, or left undissected as a bulk sample and all groups processed for RNAseq. RNAseq of stratified layers provided spatial localization of expressional changes within defined tissue regions, including angiogenesis, inflammation, and matrix remodeling.

**Results:** Different expressional profiles were observed between individual tissue layers relative to each other within a single wound group and between each individual layer relative to bulk analysis. Tissue stratification identified unique differentially expressed genes within specific layers of tissue that were hidden during bulk analysis, as well as amplification of weak signals and/or inversion of signaling between two layers of the same wound, suggesting that two layers of skin can cancel out signaling within bulk analytical approaches.

**Discussion:** The unique wound stratification and spatial RNAseq approach in this study provides a new methodology to observe expressional patterns more precisely within tissue that may have otherwise not been detectable. Together these experimental data offer novel insight into early expressional patterns and genomic profiles, within and between tissue layers, in wound healing pathways that could potentially help guide clinical decisions and improve wound outcomes.

## 1 Introduction

Skin, the largest organ of the human body, serves as an external barrier from the outside elements and provides protection from external insults (Shaw and Martin, 2009). Therefore, a wound can be defined as any injury that results in damage or disruption of the epidermal skin barrier and can often result in exposure of the deeper tissue structures to the outside elements (Shaw and Martin, 2009). Our bodies utilize a natural feedback loop to counteract and repair “open” wounds in order to “close” them and restore the epidermal barrier and protect deeper tissue structures, a process known as acute wound healing (Reinke and Sorg, 2012; Eming et al., 2014). Acute wound healing is an intricate compilation of diverse signaling cascades continuously providing both local and systemic feedback to ensure appropriate progression through the phases of wound healing (Reinke and Sorg, 2012; Eming et al., 2014). Acute wound healing involves multiple cell/tissue types and an intricate balance of dynamic molecular signaling cascades that are continuously changing as the wound tissue evolves. Thus, the ultimate goal of acute wound healing is to reestablish anatomical and functional homeostasis of the injured tissue.

To this day, there is no singular wound care intervention proven most effective for all wounds. However, application of a diverse array of wound dressing materials are often utilized and considered the mainstay of wound care (Dreifke et al., 2015; Hodge et al., 2022). One innovative therapy, that is, widely utilized clinically is negative pressure wound therapy (NPWT), which been shown to modulate the wound healing response for a number of applications, including skin grafting, surgical, traumatic, burn, orthopedic, and diabetic wounds (Argenta and Morykwas, 1997; Morykwas et al., 1997; Agarwal et al., 2019; Norman et al., 2022). NPWT involves the insertion of an open-reticulated biomaterial foam dressing into the wound, such as the polyurethane-derived GranuFoam™, coverage with a semi-permeable adhesive film, and application of subatmospheric pressures (typically 125 mmHg), via a vacuum pump, to mechanically compress the foam and subsequently decrease the wound site volume (Morykwas et al., 2006).

Overall, NPWT is well known for its ability to modulate wound healing, with clinically documented outcomes of increased rates of neovascularization, matrix production, and granulation tissue deposition, paired with augmentation of the immune response and decreases in bacterial burden (Morykwas et al., 2006; Normandin et al., 2021). A number of suggested mechanisms are associated with how NPWT exerts its effects, including the mechanical decrease of interstitial pressure via fluid egress from the wound site, the enhancement of tissue perfusion and oxygenation, and induction of mechanotransductive or foreign body responses to promote granulation tissue production (Morykwas et al., 2001; Morykwas et al., 2006; Agarwal et al., 2019; Hodge et al., 2021; Normandin et al., 2021). Additionally, recent alterations to the NPWT system have been investigated including the use of different foam materials and application of instillation devices, which has further increased the widespread implementation of NPWT as an adjuvant therapy for a variety of tissue ailments (Kim et al., 2020). Notably, the application of NPWT has also demonstrated the capacity to prepare surgical site tissue with improved grafting outcomes and decreased long-term scarring (Gupta, 2012; Webster et al., 2014). Thus, NPWT can potentially modulate the wound healing response in a number of ways and for a number of different tissue types. Yet, the dynamic mechanism(s) of how NPWT exerts its lasting effects within defined temporal and spatial confinements, remains unresolved. More specifically, it is not fully understood how different NPWT iterations and dressing material properties modulate the initial wound healing response, how these initial responses have resonating/cascading effects on wound healing several days later, what specific cells and signaling process are involved acutely, and where the key cell populations involved in the signaling are localized within the wound tissue.

The interconnected and overlapping nature of wound healing results in a complex array of molecular signaling cascades that varies amongst wound types. Notably, the topographical, architectural, and biological profile of the wound extracellular matrix (ECM) is known to modulate the wound healing process (Reinke and Sorg, 2012; Eming et al., 2014). Similarly, initial interactions between cellular populations and wound dressings properties are critical to the overall progression of the regenerative process, with different material compositions and structural formulations known to modulate cellular activity and gene expression profiles (Thevenot et al., 2008; Boehler et al., 2011; Andorko and Jewell, 2017; Jang et al., 2019). Thus, understanding how interventional wound therapies are capable of modulating wound healing at a molecular level within specific, defined regions of tissue remains a critical area of research for advancing wound therapies. Moreover, investigative new technologies to improve our current understanding of the physiological wound response will ultimately aid in the advancement of effective and targeted wound therapies. To date, wound analyses have exclusively evaluated gene expressional profiles of whole wound isolates/biopsies that compile all/multiple layers of skin tissue into one bulk sample set (Derrick and Lessing, 2014; Ma et al., 2016; Brownhill et al., 2021). Although this methodological processing has yielded large amounts of valuable information, it is limiting in its current form due to the diversity of signaling within different layers of wound tissue.

In this methodological study, a “proof-of-concept” design for a new *in vivo* porcine model is developed for screening acute wound healing interactions with different treatments and dressing materials. Due to the acute nature and interest in understanding initial wound responses, an incisional model was utilized. To provide context within this study NPWT (i.e., Granufoam™ with 125 mmHg vacuum) was utilized as the treatment group and compared to untreated control healing (i.e., no dressing insert or vacuum) within 8 h. Uniquely, this new methodology allows for the capacity to assess both the temporal and spatial components of wound healing within defined regions of the wound tissue due to the unique design, processing, and analyses. Whereas previous techniques utilize “bulk” biopsies and explanted tissue samples to perform gene expression analysis on entire wound samples, this study stratifies the skin into its three component parts, the epidermis, dermis, and subcutaneous layers. Thus, our new methodology demonstrates the capacity to potentially differentiate within sample sets and perform inter- and intra-skin layer comparisons with multivariate analyses, which offers a new perspective to view wound healing. NPWT has been shown to effect a number of processes; however, there is still much to be understood into when, where, and how it acts on specific cell populations within wounds in order to further advance its therapeutic benefits. We hypothesized that a number of critical wound healing pathways traditionally seen are likely coming from a distinct layer of skin tissue, whereas there may also be never before seen acute signaling pathways revealed due to being previously “washed out” by other layers during “Full/Bulk” tissue analysis.

## 2 Materials and methods

### 2.1 Animals

Animal studies were approved by the University of Kansas Medical Center (KUMC) Institutional Animal Care and Use Committee (IACUC) under animal care and use protocol (ACUP) #2019-2535. Two female 4-month-old miniature Yucatan pigs weighing 30–40 kg were procured from Sinclair Bio-resources (Auxvasse, MO), and allowed to acclimate for 14 days in an AAALAC accredited facility at KUMC. Animals were provided with food, water, and social enrichment *ad libitum*.

### 2.2 Surgery and sample collection

Surgeries were performed on each animal on separate days. Animals were placed under general anesthesia and ophthalmic lubricating ointment was placed to protect the eyes. The animals were prepped with three alternating scrubs of betadine and alcohol. A sterile surgical drape was placed over the animal, except for where the surgical procedure would be carried out. A custom 3D-printed acrylonitrile butadiene styrene stencil was prefabricated and used to appropriately mark the incision sites and dressing borders for the animals in a consistent manner prior to surgery. Additionally, custom designed scalpel guides were prefabricated that allowed for control of incisional depth and a double-scalpel guide was used to control the width and depth of the wound explants. Incisions were cut to a depth of 1.5-cm and a length of 2-cm. A 4 × 4 incisional array was inflicted, with the four columns representing 0-, 2-, 4-, and 8-h timepoints of explantation, and the four rows representing (dorsal to ventral) control (no dressing insert), Owen’s Rayon, GranuFoam™ (Kinetic Concepts Inc. [KCI] an Acelity company, San Antonio, TX), and polycaprolactone electrospun mesh dressings (Supplementary Figure S1). Only the 0-h and 8-h GranuFoam™ and control wound samples are discussed in this study to demonstrate “proof-of-concept”. A 2-cm × 2-cm × 0.5-cm (thick) piece of each of the three respective dressing materials were inserted in the appropriate wounds for each timepoint. Followed by placement of a Prevena foam over the top of each wound column/timepoint to bridge the dressing materials. The Prevena foams were connected to a vacuum system and set at a constant 125 mmHg for the duration of each listed timepoint. The right side of each animal received vacuum, whereas the left side of each animal served as a non-vacuum control but did have a Prevena placed over the top of each wound column. With the exception being the time 0-h columns, which were immediately processed.

**FIGURE 1:**
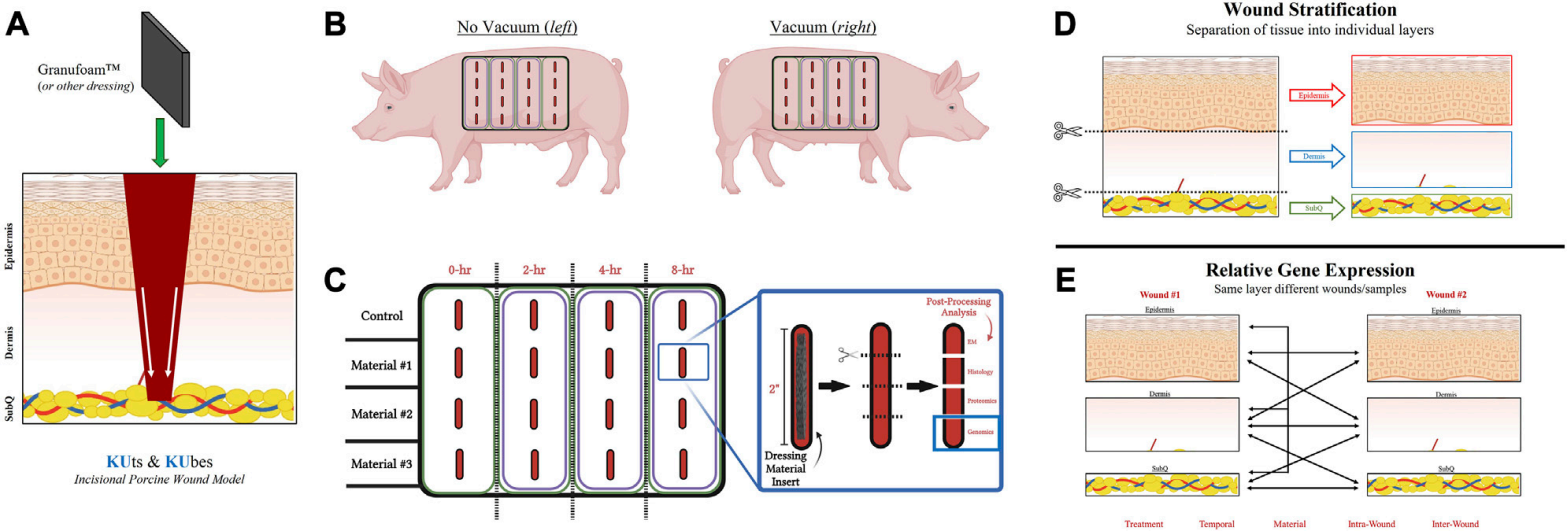
KUts and KUbes Incisional Porcine Model Experimental Design. **(A)** A schematic overview of the incisional porcine wound model depicting the incision depth and insertion of dressing material. **(B)** Overall layout of the 4 × 4 array of incisions on each side of the animals, with one side receiving NPWT. **(C)** Demonstration of the tissue processing and how wound explants would be analyzed. Different materials were inserted into different rows on the animals and at each respective timepoint the column was collected as a strip of tissue, cut into four pieces to separate out the four wounds within each strip and them each of the four wounds were further subdivided into four pieces for analysis (EM = electron microscopy). **(D)** Depiction of the stratification process of each wound explant, separating the “Full/Bulk” wound into three layers, epidermis, dermis, and subcutaneous. **(E)** A depiction of some of the multivariate analyses/comparisons that can be performed with the stratification processing technique.

At each respective timepoints, the double-scalpel guide was used to cut one long strip (entire wound column) and the predetermined depth of 2 cm. Wound explant was immediately processed, and the four treatment groups (rows) were separated, followed by further separation of each individual sample (row). Each 2-cm incisional wound was immediately processed in the operating room by a surgical assistant and cut into four equally-sized sections (5-mm thick) to allow for four different analytical approaches, including RNAlater (Sigma-Aldrich) preservation for future genomic experiments (Figure 1). The newly inflicted wound on the animals, due to wound explants, were packed with surgical gauze and compressed by hand until bleeding ceased. For RNA sample processing, wound samples were bisected in-line with the incision to generate two mirror halves of the wound and refrigerated at 4 °C in RNAlater for 24 h, followed by storage at −80°C. Animals were euthanized while under deep general anesthesia via exsanguination. A photographic overview of the experimental surgical procedure is depicted in Supplementary Figures S1A–K.

### 2.3 Processing and tissue stratification

RNAlater preserved wound samples were stratified into four groups for each wound, Full/Bulk, epidermis, dermis, and subcutaneous. First, a clean, RNAase-free workspace was created to process the tissue samples. Next, a tub of ice was prepared, an aluminum/metal sheet was placed on top of the ice and washed with 100% isopropanol followed by RNase away. All equipment and supplies were also cleaned with 100% isopropanol and RNase away. For processing, the previously bisected wound samples (two equal mirror halves of same wound) were thawed from −80°C on ice and removed prior to complete thaw so that the tissue was still hard but RNAlater had melted. Next, a Dermatome blade was used to dissect each layer from one-half of each wound sample. The remaining half was processed as a Full/Bulk wound and used to serve as the standard processing technique. Samples were immediately transported to the Genomics Core facility in fresh RNAlater (on ice) for RNA processing and isolation.

### 2.4 RNA isolation

**Epidermis and Subcutaneous Layers**. Epidermis and subcutaneous isolates from the pig wound channel were suspended in 1 ml of 4°C Invitrogen Trizol Reagent (Fisher Scientific 15-596-026) in a 5 ml tissue culture tube on ice. The tissue was homogenized using a Power Gen 35 Tissue Homogenizer employing a medium shark’s tooth probe—6 mm diameter (Fisher Scientific) for 20 s on ice. The 1 ml lysate is transferred to a Andwin Scientific 5Prime Phase Lock Gel—Heavy 2 ml tube (Fisher Scientific) and the Phase Lock gel purification is performed according to manufacturer’s protocol until the separation centrifugation of the aqueous phase and organic phase is complete. The aqueous phase is transferred to a microcentrifuge tube and the volume is determined. An equal volume of 70% ethanol in nuclease free water is added to the aqueous phase. The ethanol adjusted aqueous phase (with guanidinium salts) is applied directly to a RNeasy Mini kit (Qiagen 74104) purification column and the RNA Clean-up protocol is performed using an on-column DNase treatment with the RNase-Free DNase Set (Qiagen 79254) according to manufacturer’s instruction. DNase treated RNA isolates are quality controlled and quantified using a TapeStation 4200 running the RNA ScreenTape assay (Agilent Technologies). RNA Integrity Number (RIN) of ≥8.0 are targeted for proceeding to stranded mRNA library preparation.

**Dermis and “Full/Bulk” Layers**. Dermis and full tissue isolates (with skin) from the pig wound channel were suspended in 500ul of 4°C Invitrogen Trizol Reagent (Fisher Scientific 15-596-026) in a 5 ml tissue culture tube on ice. The tissue was homogenized using a Power Gen 35 Tissue Homogenizer employing a medium shark’s tooth probe—6 mm diameter (Fisher Scientific) for 20 s on ice. The lysate was removed to a new 5 ml tissue culture tube on ice. 500ul of additional chilled Trizol Reagent was added to the remaining un-lysed tissue (mainly skin) and homogenized an additional 20 s on ice leaving ∼10% of the original skin undisrupted. The lysate was pooled with the first lysate for 1 ml total. The 1 ml lysate is transferred to an Andwin Scientific 5 Prime Phase Lock Gel—Heavy 2 ml tube (Fisher Scientific) and the Phase Lock gel purification is performed according to manufacturer’s protocol until the separation centrifugation of the aqueous phase and organic phase is complete. The aqueous phase is transferred to a microcentrifuge tube and the volume is determined. An equal volume of 70% ethanol in nuclease free water is added to the aqueous phase. The ethanol adjusted aqueous phase (with guanidinium salts) is applied directly to a RNeasy Mini kit (Qiagen 74104) purification column and the RNA Clean-up protocol is performed using an on-column DNase treatment with the RNase-Free DNase Set (Qiagen 79254) according to manufacturer’s instruction. DNase treated RNA isolates are quality controlled and quantified using a TapeStation 4200 running the RNA ScreenTape assay (Agilent Technologies). RNA Integrity Number (RIN) of ≥8.0 are targeted for proceeding to stranded mRNA library preparation.

### 2.5 RNA library prep

**Tecan Universal Plus mRNA-Seq with NuQuant**. The stranded mRNA-Seq was performed using the Illumina NovaSeq 6000 Sequencing System at the University of Kansas Medical Center—Genomics Core (Kansas City, KS). Quality control of the total RNA isolates was completed using the Agilent TapeStation 4200 using the RNA ScreenTape Assay kit (Agilent Technologies 5067-5576). Total RNA (500 ng) was used to initiate the library preparation protocol. The 249 RNA isolates representing epidermis, dermis, subcutaneous and full tissue were randomized and divided into 11 sets of samples for library preparation. The total RNA fraction was processed by oligo dT bead capture of mRNA, fragmentation of enriched mRNA, reverse transcription into cDNA, end repair of cDNA, ligation with the appropriate Unique Dual Index (UDI) adaptors and strand selection and library amplification by PCR using the Universal Plus mRNA-seq with NuQuant library preparation kit (Tecan Genomics 0520-A01).

Validation of each sample set of library preparations was performed using the D1000 ScreenTape Assay kit (Agilent Technologies 5067-5582) on the Agilent TapeStation 4200. Concentration of each library was determined with the NuQuant module of the library prep kit using a Qubit 4 Fluorometer (ThermoFisher/Invitrogen), Libraries were normalized to 4 nM concentration and pooled as a sample set. Each multiplexed sample pool set was quantitated, in triplicate, using the Roche Lightcycler96 with FastStart Essential DNA Green Master (Roche 06402712001) and KAPA Library Quant (Illumina) DNA Standards 1-6 (KAPA Biosystems KK4903). Using the qPCR results, each sample pool set was adjusted to 1.9 nM. All 11 normalized sample pool sets were combined for a final normalized pool to perform multiplexed sequencing.

The normalized and pooled libraries were denatured with 0.2N NaOH (0.04N final concentration) and neutralized with 400 mM Tris-HCl pH 8.0. The pooled libraries were diluted on sequencer to 380 pM prior to onboard clonal clustering of the patterned flow cell using the NovaSeq 6000 S4 Reagent Kit v1.5 (200 cycle) (Illumina 20028313). The 2 × 101 cycle sequencing profile with dual index reads is completed using the following sequence profile: Read 1–101 cycles x Index Read 1–8 cycles × Index Read 2–8 cycles x Read 2–101 cycles. Following collection, sequence data are converted from. bcl file format to “fastq” file format using bcl2fastq software and de-multiplexed into individual sequences for data distribution using a secure FTP site or Illumina BaseSpace for further downstream analysis.

### 2.6 Differential gene expression

RNA-Sequencing was performed at a strand specific 100 cycle paired-end resolution, in an Illumina NovaSeq 6000 sequencing machine (Illumina, San Diego, and CA). The analysis was performed on two animals (biological duplicate) with samples obtained from different regions subject to different treatments from each animal as described in the text. The samples were multiplexed in an S1 flow-cell, resulting between 33.6 and 57.9 million reads per sample. The read quality was assessed using the FastQC software. On average, the per sequence quality score measured in the Phred quality scale was above 30 for all the samples. The reads were mapped to the *sus scrofa* genome (Sscrofa11.1; Ensembl release 105) using the STAR software, version 2.6.1c. On average, 95% of the sequenced reads mapped to the genome, resulting between 31.8 and 54.8 million mapped reads per sample, of which on average 92% were uniquely mapped reads. Transcript abundance estimates were calculated using the featureCounts software (Liao et al., 2014). Expression normalization and differential gene expression calculations were performed in DESeq2 software to identify statistically significant differentially expressed genes (Love et al., 2014). The samples were analyzed as matched paired samples. The significance *p*-values were adjusted for multiple hypotheses testing by the Benjamini and Hochberg method establishing a false discovery rate (FDR) for each gene. The differential expression analysis of high-throughput data for this study was performed in R using the DESeq2 package. The DESeq2 package performs an independent filtering of the results by default and therefore requires minimal prefiltering. The input to the software was the raw count matrix with genes with less than one count per million in any of the samples removed. The DESeq2 software performs a median of ratios normalization that accounts for sequencing depth and RNA composition.

### 2.7 Bioinformatic and statistical analysis

Due to the nature of this study being a ‘pilot study’ only two animals were utilized. Global gene expression was performed for control wounds at 8-h (no dressing insert and no vacuum) and for NPWT-treated wounds at 8-h (Granufoam™ insert and 125 mmHg vacuum), relative to baseline expression at 0-h control (no dressing insert and no vacuum). Additionally, a second set of analyses was performed for differentially expressed genes (DEGs) for 8-h NPWT-treated wounds relative to 8-h control wounds to assess the effect of NPWT treatment relative to the control response. All analyses were performed for each layer relative to its analogous layer (e.g., epidermis relative to epidermis), in addition to the 0-h control “Full/Bulk” wounds. DEGs were investigated by with Ingenuity Pathways Analysis (IPA) software (Qiagen; Ingenuity Systems Inc.; CA and United States). IPA identified canonical pathways, diseases and functions, upstream regulators, and gene networks that were most significant to the relative gene expression profiles for each set of wounds and categorized the DEGs into specific pathways. Heatmaps, graphs, gene clusters, and network webs were automatically generated within IPA and used to visually depict changes in DEGs. A false discovery rate (FDR) below 0.05 was always utilized to assess for DEGs, and was the only threshold qualification when initially assessing for the heterogeneity in global gene expression. Additional thresholding was performed on subsequent pathway analyses, including a minimum -log (*p*-value) of 1.3, which is equal to *p* < 0.05, and an experimental fold change (FC) of 1.5. The additional thresholding was performed for generation of significant canonical signaling pathways, heatmap generation, and top regulatory network analyses. When performing the intra-wound comparison between the different stratified layers to assess for hidden DEGs within the inflammatory, angiogenic, and matrix remodeling pathways, an FDR of 0.05 and a -log (*p*-value) of 1.3 was used to threshold for significantly altered genes.

## 3 Results

### 3.1 KUts and KUbes incisional porcine model experimental design

A schematic overview of the incisional porcine wound model can be found in Figure 1. Incisional wounds were created with a depth that penetrated all three skin layers and into the subcutaneous region, followed by insertion of dressing materials into the wounds to permit tissue interactions with the dressings at each layer (Figure 1A). The incisional model was performed as an array on each side of the animal, with different dressing materials making up the rows and different timepoints (0-, 2-, 4-, and 8-h) making up the columns (Figures 1B, C). However, only the 0- and 8-h wound groups were utilized in this study. Additionally, only the genomic analysis was performed in this “proof-of-concept” study due to its higher sensitivity to detect changes in early wound healing pathways (i.e., within 8 h). A complete photographic depiction of the surgical operations can be found in Supplementary Figures S1A–K. Upon excision of the wounds at each respective timepoint, tissue was processed for downstream analysis accordingly. For RNA analysis, samples were dissected/stratified into their three separate skin layers and subsequently processed (Figures 1D, E). The stratification of the wounds allowed for several different intra- and inter-related analyses that were dressing, temporal, or treatment dependent, as well as untreated control wounds to understand physiological signaling (Figure 1E).

### 3.2 Heterogeneity in global gene expression profiles between wound layers

**FIGURE 2:**
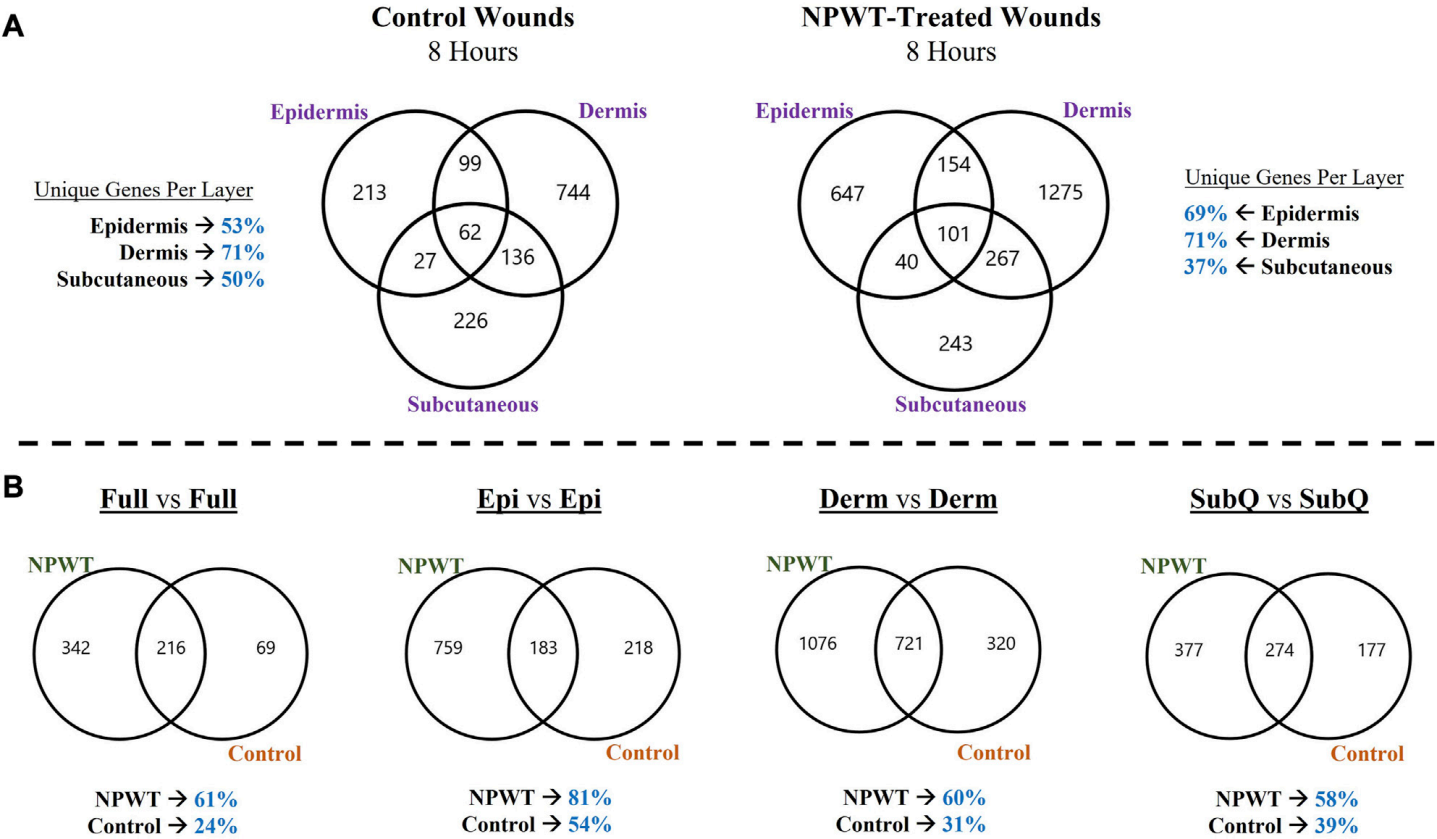
Heterogeneity in Global Gene Expression Profiles Between Wound Layers. **(A)** Graphical depiction of differentially expressed genes (DEGs) for Control (*left*) and NPWT-treated (*right*) wounds that were stratified and separated into each specific layer and overlayed. “Layer Specific Genes” denotes the percentage of DEGs that were uniquely expressed in that layer and none of the other layers. **(B)** Direct comparison of unique DEGs for within each layer for either NPWT-treated or control wounds. The percent of uniquely expressed DEGs for each condition is listed below each respective graph. Of note, the graphs do not depict directionality, only overlap in DEGs that have met the predefined threshold of an FDR less than 0.05.

To depict the overall heterogeneity of molecular signaling that occurs within the different layers of skin tissue during wound healing, the differentially expressed gene (DEGs) profiles were overlayed and evaluated for uniqueness to specific layers and/or NPWT treatment (NPWT is defined as wounds treated with GranuFoam™ and 125 mmHg vacuum). Of note, the graphs do not depict directionality, only overlap in DEGs that have met the predefined threshold of an FDR less than 0.05 (Figures 2A, B). First, an intra-comparison within a single wound between tissue layers were evaluated for both control healing (no dressing insert and no vacuum at 8-h) and NPWT-treated healing at 8 h, relative to the baseline control wound expression at 0-h, to depict relative uniqueness (Figure 2A). The dermis layer for both control and NPWT wounds exhibited the greatest proportion of DEGs uniquely associated within its layer, at 71%. Whereas the epidermis had the second most and the subcutaneous had the lowest proportion (Figure 2A). Conversely, the relative similarities and overlap between DEGs within the Full *vs*. Full and Layer *vs*. Full comparisons, for each layer, is depicted in Supplementary Figure S2 and demonstrates that there was minimal overlap of the layer analyses with the Full analyses, with the average similarity/overlap of the Layer-DEGs with the Full-DEGs coming in at <15% of the total DEGs for each Layer (Supplementary Figure S2). Subsequently, inter-comparison of control *versus* NPWT-treated wounds within each analogous layer revealed that NPWT treatment modulated the DEG profile, relative to control wounds, at 8 h 81%, 60%, and 58% of the total DEGs were unique for NPWT treatment for the epidermis, dermis, and subcutaneous regions, respectively (Figure 2B). Notably, the relative proportion of uniquely expressed DEGs in NPWT-treated and 8-h control wounds were similar in the “Full *versus* Full” comparison group, the total number of DEGs was substantially lower compared to the layer *versus* layer comparisons. The average total number of DEGs in the layer comparisons was 1130 for NPWT-treated and 631 for 8-h control wounds. On the contrary, in the “Full *versus* Full” comparison analysis, there was a >50% reduction, with only 558 DEGs for NPWT-treated wounds and 285 in the 8-h control healing wounds.

### 3.3 Stratified genomic analysis reveals temporal-spatial heterogeneity in canonical signaling pathways

**FIGURE 3:**
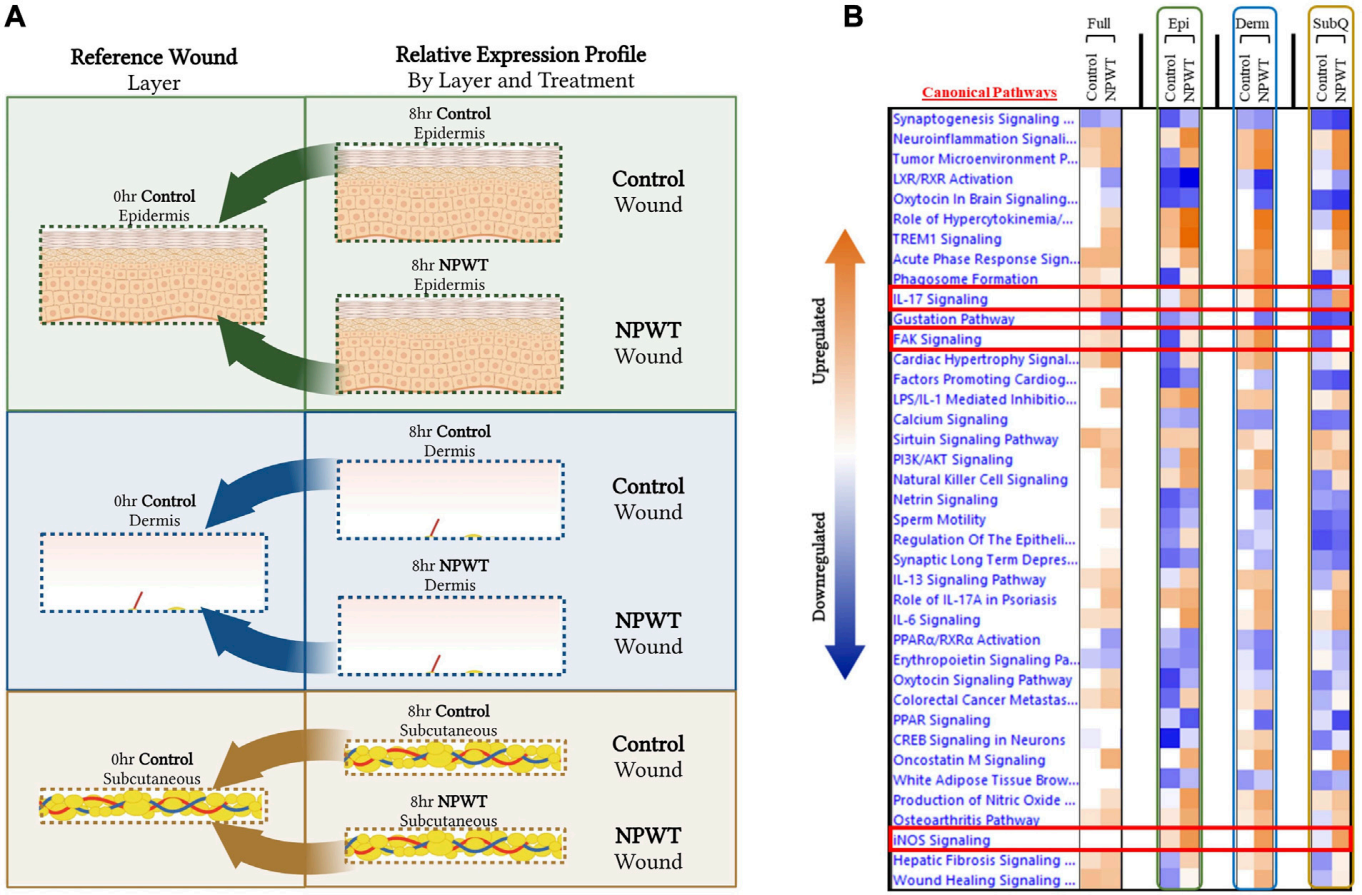
Stratified Genomic Analysis Reveals Temporal-Spatial Heterogeneity in Canonical Signaling Pathways. A schematic representation of wounds were stratified and DEGs were generated via relative expression to 0-h control wounds for both 8-h control (untreated) and 8-h NPWT-treated at each layer, including the **(A)** epidermis (*top*), dermis (*middle*), and subcutaneous (*bottom*) regions. **(B)** A heatmap of the top canonical pathways with a FDR of 0.05 and a *p*-value < 0.05. Orange depicts upregulation and blue depicts downregulation of each pathway. White denotes no significant change in pathway activity.

To provide context for the type of data that can be obtained with this methodology, a DEG comparison was performed on the progression of NPWT healing *versus* untreated control healing at the 8-h timepoint. Gene expression for both NPWT and control 8-h samples were generated relative to the 0-h baseline control wounds (Figure 3A). “Full/Bulk” wound comparisons were performed in parallel to the stratified comparisons, which include the epidermis, dermis, and subcutaneous regions of each wound in the NPWT or 8-h control groups, relative to the analogous region in the 0-h baseline control (Figure 3A). A heatmap of the top canonical pathways was generated for the “Full *versus* Full”, “Epidermis *versus* Epidermis”, “Dermis *versus* Dermis”, and “Subcutaneous *versus* Subcutaneous” comparisons (Figure 3B). When comparing the relative gene expression of the “Full/Bulk” processed wounds, there was minimal changes in expression and canonical signaling, such as that seen in the iNOS signaling pathway, or minimal difference between NPWT and control healing as seen in the IL17 signaling pathway (Figure 3B). However, stratification processing revealed that when focusing on specific regions within the wounds (e.g., epidermis *versus* epidermis), several signaling pathways are revealed to be significantly upregulated or downregulated that were previously not in the “Full/Bulk” comparisons, including inversion of signaling, that is, seen with FAK signaling or the amplification of signaling, that is, seen with iNOS (Figure 3B).

### 3.4 Tissue stratification reveals canonical signaling and top pathway regulators previously hidden within acute physiological wound healing

**FIGURE 4:**
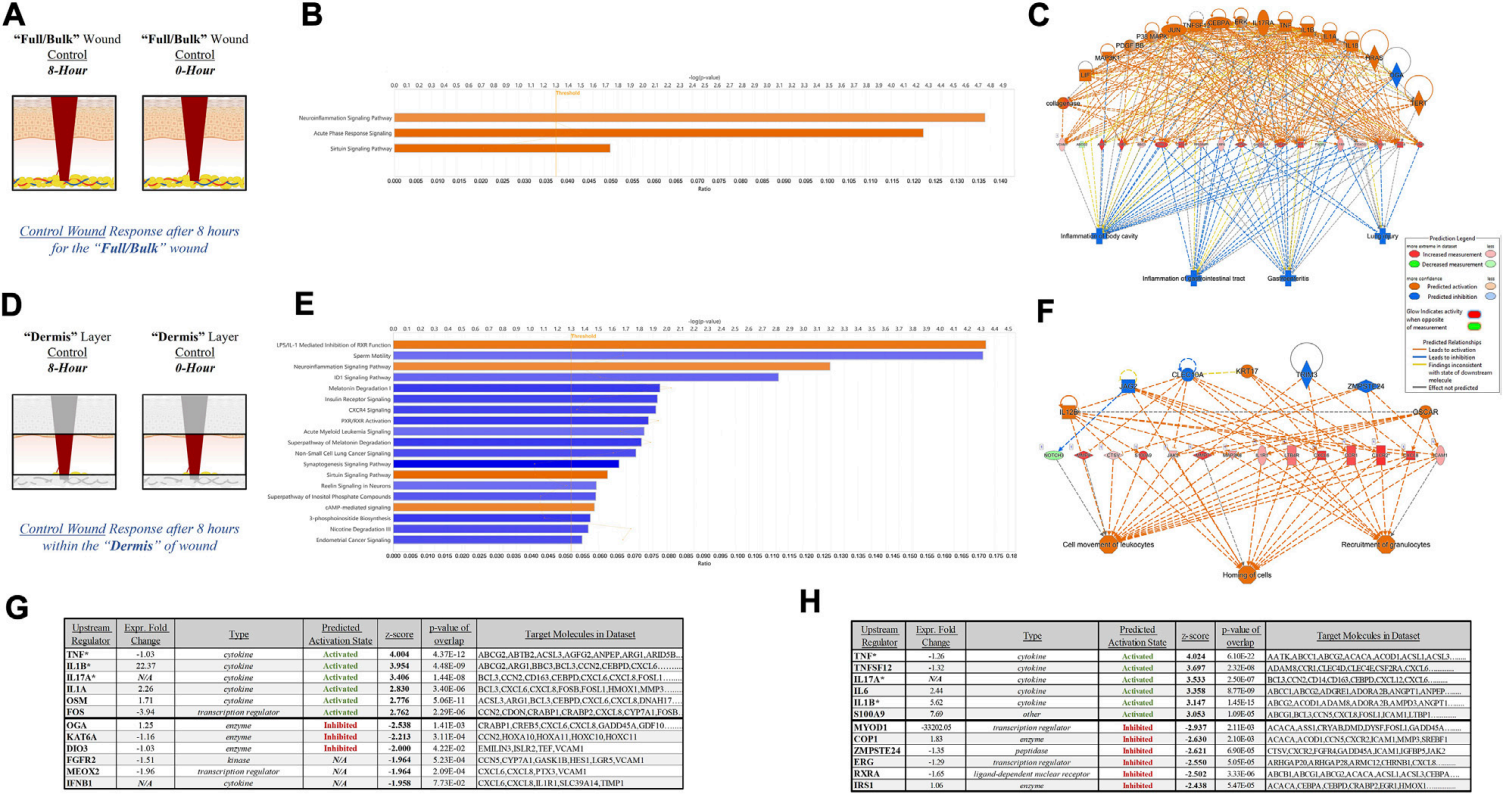
Tissue Stratification Reveals Canonical Signaling and Pathway Regulators Previously Hidden in Acute Physiological Wound Healing. **(A, D)** Schematic representations of the **(A)** “Full/Bulk” and **(D)** stratified dermis 8-h and 0-h wounds that were processed for analysis. **(B, E)** Graphical depictions of the top canonical pathways that achieved significance after thresholding, -log (*p*-value) of 1.3 and FC of 1.5. The threshold line is marked at 1.3. Color denotes fold change directionality, with activation/upregulation (*orange*) and inhibition/downregulation (*blue*). **(C, F)** The top regulator effect networks associated with the DEGs for the **(C)** “Full/Bulk” analysis and **(F)** stratified dermis analysis. **(G, H)** The top 6 upstream regulator activators and inhibitors are represented in tabular form for the **(G)** “Full/Bulk” analysis and the **(H)** stratified dermis analysis.

To further depict the increased sensitivity and specificity associated with performing gene expression analysis on stratified wound sections rather than “Full/Bulk”, we performed a direct comparative analysis of top canonical pathways and regulators significantly altered during untreated (control) healing at 8 h. Thresholds utilized to determine significance was an FDR 0.05, -log (*p*-value) of 1.3, and a FC of 1.5. When evaluating 8-h control wounds via “Full/Bulk” analysis relative to “Full/Bulk” 0-h baseline control wounds (Figure 4A), only 3 canonical pathways demonstrated a significant modulation, all of which had an FC increase (denoted by orange color), and were nonspecifically associated with inflammation. Expressional changes included the canonical pathways of neuroinflammation, acute phase response, and sirtuin signaling pathways, and the top regulatory network was associated with inhibition of gastrointestinal, lung, and body cavity inflammation (Figures 4B, C). Whereas, when evaluating the same exact 8-h control wounds via stratification of the dermis layer only (Figure 4D), 2 of the 3 same canonical pathways from the “Full/Bulk” analysis were significant, in addition to 17 additional canonical pathways, for a total of 19 significantly altered pathways, most of which exhibited a negative fold change (denoted by blue color). With the top regulatory network being associated with cell movement of leukocytes, homing of cells, and recruitment of granulocytes (Figures 4E, F). Subsequently, the top 6 upstream regulator molecules associated with either activation or inhibition (based on the z-score) were tabulated for both the “Full/Bulk” and stratified dermis analyses (Figures 4G, H). Of the 12 total markers (6-up/6-down), only 3 were shared between the “Full/Bulk” and the dermis methods, including the top activator TNF, as well as IL1B and IL17A.

### 3.5 Stratification analysis identifies and localizes key physiological wound responses altered by NPWT not previously observed

**FIGURE 5:**
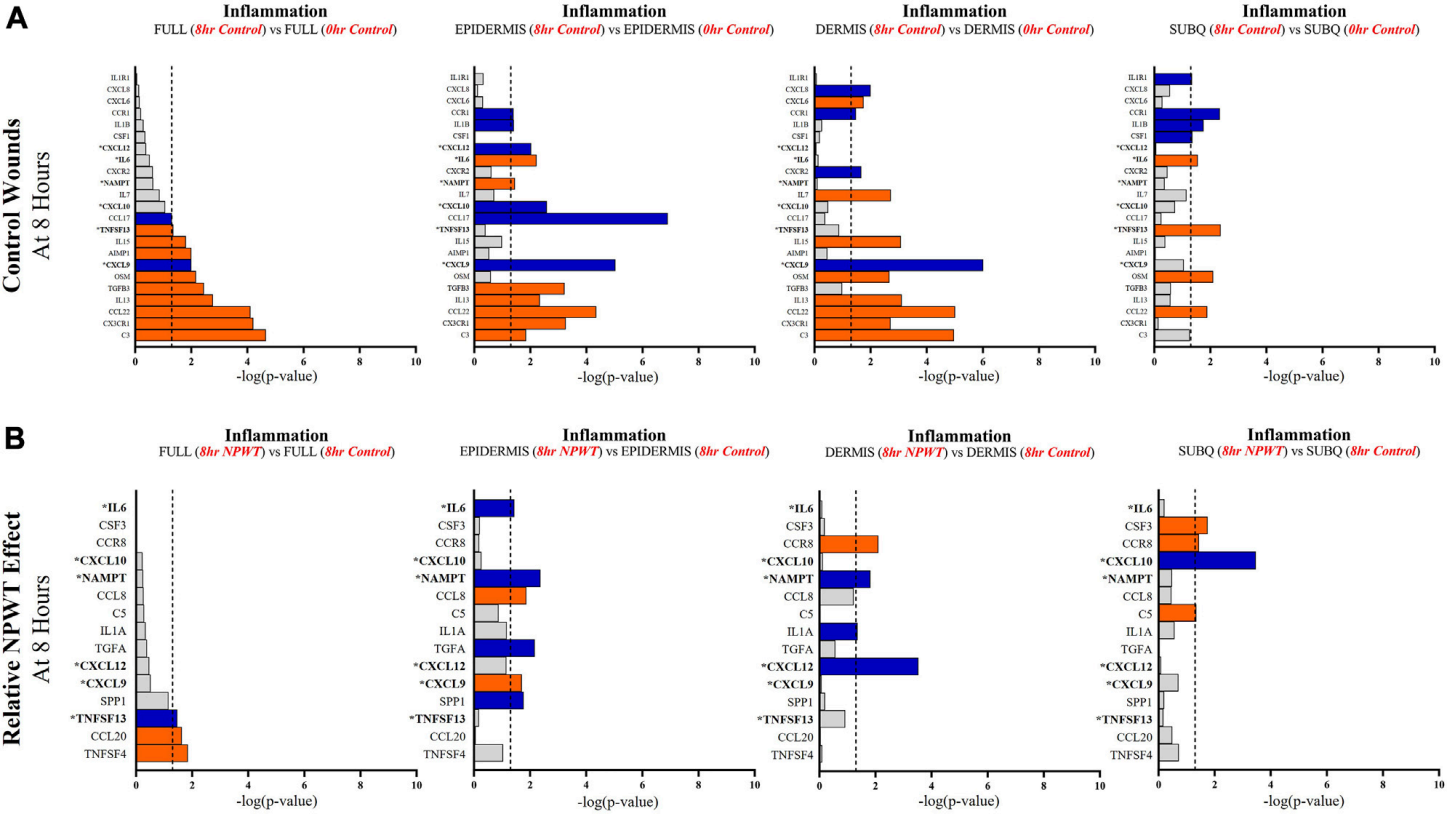
Stratification Analysis Identifies and Localizes Inflammatory Responses Altered by NPWT. An intra-wound analysis that compared DEGs associated with the inflammatory response within each layer of skin and relative to the “Full/Bulk” analyzed wounds that achieved the predetermined significance [significance determined to be a -log (*p*-value of 1.3 which equals a *p* < 0.05]. Associated DEGs were analyzed in the setting of the **(A)** control wound healing response at 8-h relative to 0-h baseline control and **(B)** NPWT-treated response at 8-h relative to 8-h control. A significance threshold line is denoted at 1.3. Size of bar denotes level of significance. Color of bars denote relative fold change directionality as either upregulated (*orange*) or downregulated (*blue*). DEGs that were common between control and NPWT wounds are “bolded” and denoted with an “*”.

**FIGURE 6:**
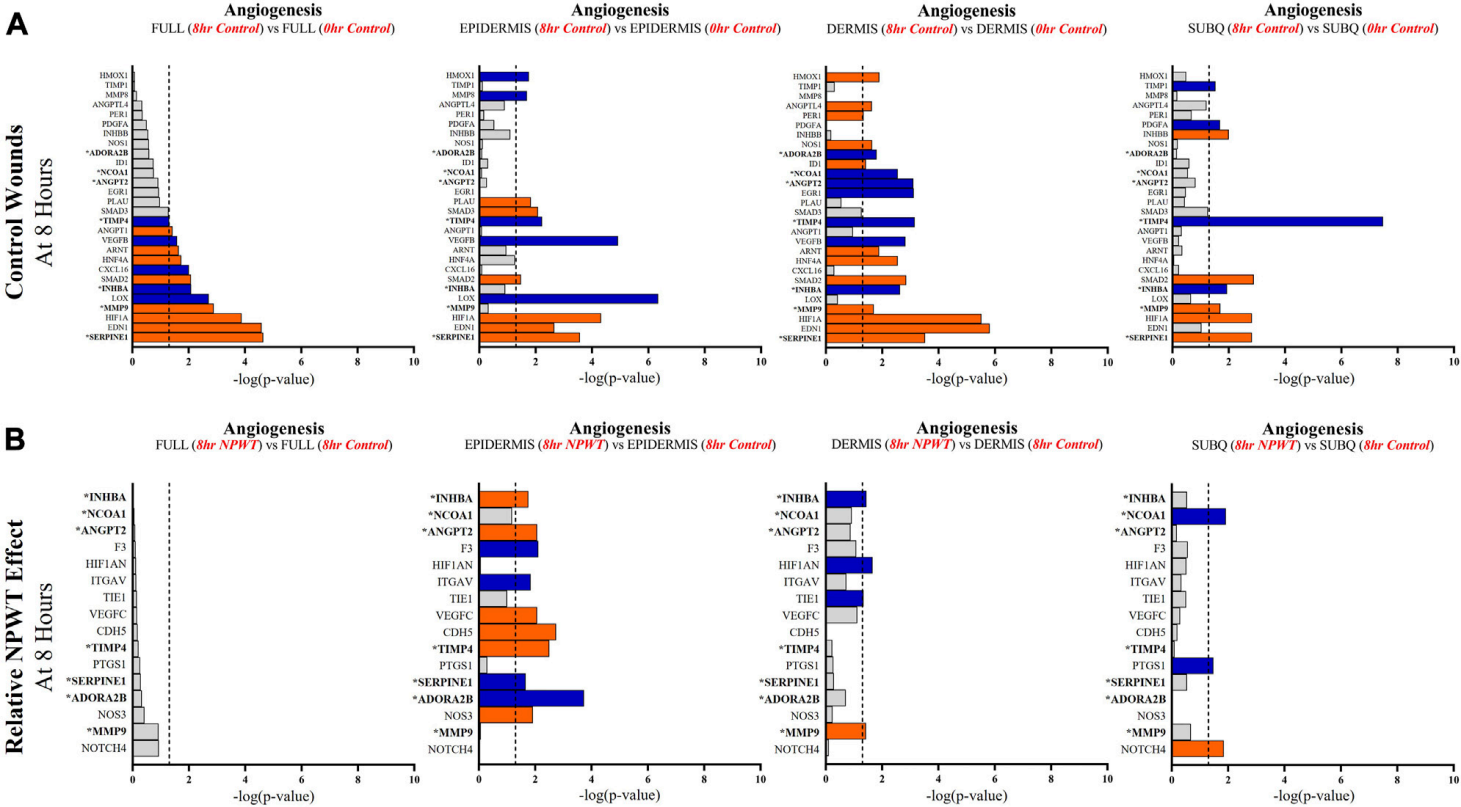
Stratification Analysis Identifies and Localizes Angiogenic Responses Altered by NPWT. An intra-wound analysis that compared DEGs associated with the angiogenic response within each layer of skin and relative to the “Full/Bulk” analyzed wounds that achieved the predetermined significance [significance determined to be a -log (*p*-value of 1.3 which equals a *p* < 0.05]. Associated DEGs were analyzed in the setting of the **(A)** control wound healing response at 8-h relative to 0-h baseline control and **(B)** NPWT-treated response at 8-h relative to 8-h control. A significance threshold line is denoted at 1.3. Size of bar denotes level of significance. Color of bars denote relative fold change directionality as either upregulated (*orange*) or downregulated (*blue*). DEGs that were common between control and NPWT wounds are “bolded” and denoted with an “*”.

**FIGURE 7:**
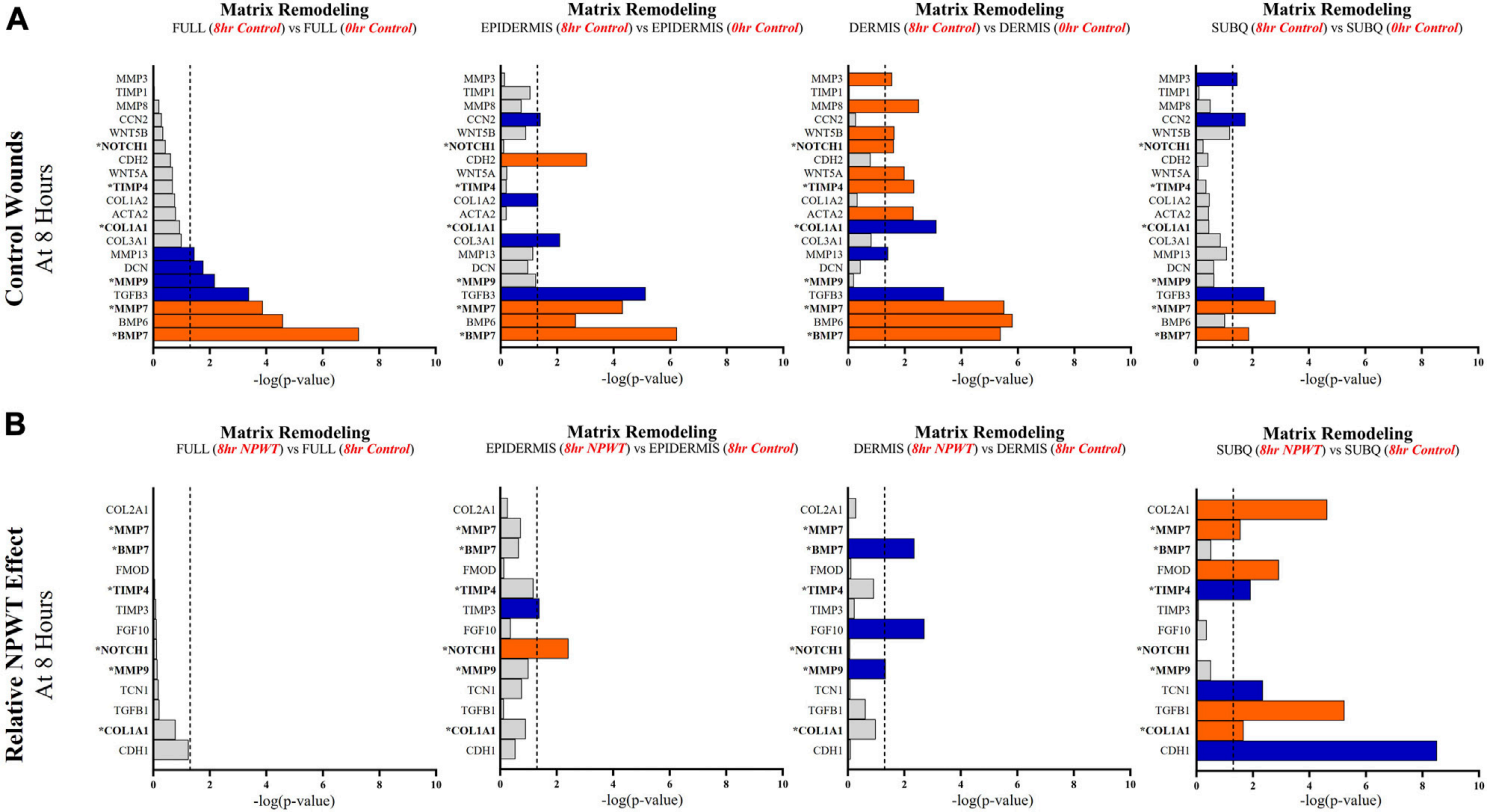
Stratification Analysis Identifies and Localizes Matrix Remodeling Responses Altered by NPWT. An intra-wound analysis that compared DEGs associated with the matrix remodeling response within each layer of skin and relative to the “Full/Bulk” analyzed wounds that achieved the predetermined significance [significance determined to be a -log *p*-value of 1.3 which equals a *p* < 0.05]. Associated DEGs were analyzed in the setting of the **(A)** control wound healing response at 8-h relative to 0-h baseline control and **(B)** NPWT-treated response at 8-h relative to 8-h control. A significance threshold line is denoted at 1.3. Size of bar denotes level of significance. Color of bars denote relative fold change directionality as either upregulated (*orange*) or downregulated (*blue*). DEGs that were common between control and NPWT wounds are “bolded” and denoted with an “*”.

Specific wound healing signaling pathways, including inflammation (Figure 5), angiogenesis (Figure 6), and matrix remodeling (Figure 7), were evaluated and DEG analysis was performed with thresholds. All genes included for each pathway passed the thresholds of an FDR of 0.05. Additionally, if a gene reached a significant -log (*p*-value) of 1.3 in at least one of the four comparative groups (Full *vs*. Full, Epi *vs*. Epi, Derm *vs*. Derm, or SubQ *vs*. SubQ), then it was included in the final comparative analysis (Figures 5). For each wound healing signaling pathway, the untreated response was first determined by evaluating the 8-h control relative to the 0-h baseline control wounds (Figures 5A–7A), followed by assessing the direct effect of NPWT via comparing the 8-h NPWT-treated relative to the 8-h control (Figures 5B–7B).

When evaluating inflammatory signaling, there were a total of 23 signaling molecules significantly altered during untreated healing, whereas when looking specifically at genes that were altered with NPWT relative to the control response (i.e., the effect of NPWT) only 15 were observed, 6 of which were similar between untreated control and NPWT treatment (Figures 5A, B). Notably, 12 of the 23 (52%) genes included in the control response were not significant in the “Full/Bulk” comparison but were within one of the three stratified layer comparisons, including IL1B, IL6, and IL7 (Figure 5A). When evaluating the effect of NPWT, only 3 of the 15 significantly altered genes were observed within the “Full/Bulk” analysis (Figure 5B).

When evaluating angiogenic signaling, there were a total of 28 signaling molecules significantly altered during untreated healing, whereas when looking specifically at genes that were altered with NPWT relative to the control response only 16 were observed, 7 of which were similar between untreated control and NPWT treatment (Figures 6A, B). Notably, 15 of the 28 (54%) genes included in the control response were not significant in the “Full/Bulk” comparison but were within one of the three stratified layer comparisons, including PDGFA, ANGPT2, and INHBB (Figure 6A). Notably, INHBA, SERPINE1, and SMAD2 are all significantly upregulated in all three stratified layers, whereas TIMP4 is significantly downregulated in all three layers. When evaluating the effect of NPWT, 0 of the 16 significantly altered genes were observed within the “Full/Bulk” analysis (Figure 6B). Interestingly, the layer with the most activity in gene expression was the epidermis, with 10 out of 16 of the genes significantly modulated, where NPWT resulted in upregulation of TIMP4 and downregulation of SERPINE1. INHBA demonstrated an inversion of signal, with an upregulation in the epidermis and a downregulation within the dermis (Figure 6B). The dermis only had 2 uniquely expressed genes, TIE1 and MMP9, whereas the subcutaneous region had 3 genes, NCOA1, PTGS1, NOTCH4.

When evaluating matrix remodeling signaling, there were a total of 20 signaling molecules significantly altered during untreated healing, whereas when looking specifically at genes that were altered with NPWT relative to the control response only 13 were observed, 6 of which were similar between untreated control and NPWT treatment (Figures 7A, B). Notably, 13 of the 20 (65%) genes included in the control response were not significant in the “Full/Bulk” comparison but were within one of the three stratified layer comparisons, including the notable genes MMP3, MMP8, COL1A1, COL3A1, TIMP1, and TIMP4 (Figure 7A). During untreated acute healing the dermis layer appeared to demonstrate the greatest upregulation of matrix remodeling activity, whereas the subcutaneous exhibited the least (Figure 7A). When evaluating the effect of NPWT, 0 of the 13 significantly altered genes were observed within the “Full/Bulk” analysis (Figure 7B). Interestingly, the subcutaneous region exhibited the greatest alteration of matrix remodeling activity after NPWT, with 8 of the 13 significantly altered genes being uniquely expressed, whereas the epidermis exhibited only 2, and the dermis only 3 (Figure 7B).

## 4 Discussion

The development of new wound dressings and therapies is an ever-growing field of research, with new targeted and personalized wound dressings for specific applications at the forefront of investigative research (Hodge et al., 2022). However, the skin is a diverse and stratified tissue comprising of diverse populations within the epidermis, papillary dermis, reticular dermis, and subcutaneous adipose (hypodermis) (Shaw and Martin, 2009). Within each stratified layer there are unique compositions of critical structures, including various degrees of lymphatics, blood vessels, sensory nerve endings, glands, and hair follicles. Additionally, each layer is composed of distinct cell populations, as well as supporting cell populations that all ultimately contribute to the processes involved in the maintenance of skin homeostasis and the wound healing response (Shaw and Martin, 2009). Therefore, a targeted approach to analyzing wounds is necessary in order to tease apart and fully understand the immense complexity of the tissue and its regenerative processes. Additionally, generating a fundamental understanding of how acute wound responses relate to long-term trajectory of wound outcomes and how acute interventions and wound dressings modulate the molecular profile of specific cells is critical to advancing targeted therapies.

One such wound therapy, that is, widely utilized is NPWT, and it offers the opportunity for customization and tailoring for specific applications, such as modulating the foam dressing material (e.g., polyurethane *versus* polyvinyl alcohol), structural properties of the dressing (e.g., pore sizes or hydrophobicity), incorporation of instillation and wound irrigation, or may be used in combination with other wound therapies or inclusion of tissue regeneration modalities. However, to date, a basic understanding of the acute temporal-spatial effects of NPWT on wound tissue at a molecular level, relative to physiological healing, is poorly understood. Clinically, we see enhancements of granulation tissue formation, modulation of inflammation, and mitigation of bacterial burden, but how NPWT globally alters specific molecular signaling pathways in both time and space to ultimately achieve these processes is not well documented. Therefore, NPWT was utilized within this methodological study to provide context for a new “proof-of-concept” *in vivo* study design and multiplex molecular analysis approach to provide new insight into NPWT molecular signaling.

In this study a new incisional porcine wound model was created to screen and standardize the processing and analysis of wound interventions in an acute setting, such as NPWT (Supplementary Figure S1). Since understanding the acute physiological responses and interactions of tissue with wound dressing inserts was desired, an incisional model was utilized to maximize the number of timepoints and dressings that could be compared in parallel. Additionally, as we know, early signaling is critical for the proper progression to later downstream signaling pathways, yet there is very little literature on acute wound signaling and genomics. By inflicting a wound with a depth that penetrated through the subcutaneous layer, we were also able to analyze all three major layers of skin. Each wound was bisected, with one-half serving as the “Full/Bulk” analysis, that is, standard for current wound genomic studies, and the other half was stratified into the 3 layers of the skin to allow for direct comparisons within the same wound (Figures 1D, E). The goal of this study was to depict, in principle, the advantages of this methodology, and thus only a comparison between NPWT-treated (Granufoam™ with 125 mmHg vacuum) and untreated control healing (no dressing insert or vacuum) at 8 h was evaluated.

When assessing for global gene expression changes in control and NPWT-treated wounds, significant diversity was observed in expressional profiles within each layer of skin within the same wounds, with each layer of the control wound exhibiting over 50% of their gene expression unique to that respective layer, with the dermis having the greatest number of unique DEGs at 71%. The NPWT-treated wound exhibited a similar pattern of gene expression diversity (Figure 2A). Thus, the previously utilized “Full/Bulk” approach to wound genomics is an amalgamation of the entire wound healing response within the tissue, however it does not provide any spatial information as to where unique signaling is coming from.

Further extrapolation of the wounds’ gene expression profiles was performed via direct comparison of NPWT-treated and control healing at each layer, which depicted extensive heterogeneity in signaling. However, when comparing the global gene expression of the stratified layer analyses *versus* the “Full/Bulk”, there was over a 50% decline in total number of DEGs in the “Full/Bulk” group (Figure 2B). This is likely due to competing signals between layers within a wound and dilution of signaling from other layers. This phenomenon is demonstrated in Figure 3, where specific canonical signaling pathways that are minimally altered within the “Full/Bulk” analysis are newly revealed within the stratified analysis or exhibit amplification and/or inversion of signal, which are highlighted with the IL17, FAK, and iNOS signaling pathways (Figure 3B). This suggests that if there is upregulation in one layer of skin and downregulation in another layer, then these two signals could cancel out and result in no signal present when performing a “Full/Bulk” tissue analysis, resulting in a loss in pertinent spatial information regarding the wound healing process. To the authors’ knowledge, this is the first depiction of the diverse temporal-spatial modulation of signaling pathways for acute physiological healing in the context of NPWT in such a manner. Thus, the sensitivity and specificity of “Full/Bulk” analytical approaches is drastically hindered due to competing signals within different layers of skin tissue that may result in key hidden signaling pathways.

Next, to further demonstrate the dilution of key signaling pathways in traditional “Full/Bulk” processing and subsequently examine the increased specificity associated with our stratified approach, the authors investigated changes in the top changes in canonical and regulatory pathways within untreated control wounds and compared the traditional “Full/Bulk” approach to changes solely within the dermis to reveal hidden signaling. As expected, there was over a 6-fold increase in significantly altered canonical signaling pathways when performing the stratified analysis with just the dermis, relative to the “Full/Bulk (Figures 4B, E). Moreover, the “Full/Bulk” signaling was less specific and broadly associated with inflammatory activity in a variety of tissues and the top regulatory network was much more complex in nature (Figure 4C). Whereas the dermis stratified analysis revealed 17 hidden canonical pathways, and when assessing the top regulatory network correlated with the dermal gene activity, a more concise and focused network tree was observed (Figures 4E, F). Specifically, the top regulatory network in the dermis was found to be associated with leukocyte and phagocyte migration into the dermis, an expected key wound healing event that occurs in the dermis within the first 24 h (Figure 4F). Unsurprisingly, 3 of the top upstream regulators found in both analyses are IL1B, IL6, and TNF, all of which have broad activity functions during wound healing and the inflammatory response. However, a top upstream regulator found within the dermis that was not noted within the “Full/Bulk” samples is S100A9, a key damage associated molecular pattern (DAMP), that is, found in myeloid cells, such as macrophages, and a known modulator of inflammatory activity, which is recently being investigated for a potential role in re-epithelialization (Figure 4H) (Kerkhoff et al., 2012). Thus, further investigations into physiological regulators may reveal critical prognostic markers for specific wound types (e.g., burns and diabetic ulcers) and potential targets for optimizing current and future wound therapies.

Subsequently, a direct intra-wound and inter-wound comparison between skin layers of DEGs associated with specific wound healing pathways was evaluated, including inflammation, angiogenesis, and matrix remodeling. Genes included in the analysis for each pathway were derived from a larger gene pool predefined to be associated with each pathway. Thresholding was used to denote DEGs that reached a -log (*p*-value) of 1.3 denoted as significant. However, a DEG had to only reach significance in one of the four groups to be included within the analysis, thus, hidden signaling in a single layer could be identified and its relative expression compared to other layers. This was done for both control wounds at 8-h, relative to the 0-h baseline control, and as a direct comparison of the effect of NPWT at 8-h, relative to 8-h control. It is important to note that the NPWT-treated profile is not the absolute/global profile of the wounds but relative to the 8-h control wounds to assess for the direct effect of NPWT. This approach revealed that over 50% of the significantly expressed DEGs in each of the three pathways being hidden within the “Full/Bulk” analysis for the control group, and over 80% for the NPWT-treated group. Further demonstrating a significant loss in spatial gene expression information in acute wound healing with the “Full/Bulk” analysis.

Further evaluation revealed that several keys signaling markers in all three pathways were being differentially expressed in a specific layer of skin tissue that was not observed in “Full/Bulk” analysis. For example, in the 8-h control groups, the inflammatory markers IL6 and IL1B were only differentially expressed in the epidermis and subcutaneous regions, whereas IL7 and CXCL6 were only expressed in the dermis. Moreover, the epidermis and dermis appear to have similar inflammatory expressional profiles overall, whereas the subcutaneous region had a much less extensive inflammatory role in acute wound healing. Notably, CXCL6 has recently been investigated as a potential biomarker for wound healing in the context of diabetic wounds (Eming et al., 2010; Wang et al., 2019), suggesting a potential origin of this factor may be within the dermis, although this was not a diabetic model and this would need further investigation. Similarly, within the NPWT wounds, the inflammatory marker IL6 was only significantly altered (downregulated) within the epidermis, IL1A was only significantly altered (downregulated) within the dermis, and CSF3 (granulocyte colony-stimulating factor) was only significantly altered (upregulated) within the subcutaneous region (Figures 5A, B). Interestingly, NPWT appeared to exhibit, in the acute setting, an anti-inflammatory stimulus relative to untreated control wounds. Thus, the stratified approach allows for the targeted observation of the inflammatory profile of wounds within defined regions of tissue and the opportunity to assess inflammatory markers for prognostic applications.

Angiogenic and matrix remodeling signaling profiles exhibited similar patterns, with substantial heterogeneity in signaling between layers and 0 differentially expressed genes within the “Full/Bulk” sample groups after NPWT treatment for either pathway. Notably, within the angiogenic pathway, several key markers appear to not be significantly expressed in the acute setting, including VEGFA, NOS1, or FGF2, markers considered to promote angiogenesis in wound healing. Thus, these critical markers may receive input from a different regulatory marker initially before becoming upregulated in wounds, during both untrated control wounds and with NPWT. Interestingly, significant gene expression associated with angiogenesis is occurring within the epidermis, rather than the dermis, including NOS3 (eNOS), CDH5 (VE-Cadherin), and ANGPT2 (Angiopoietin 2). Additionally, there is a direct inversion in signaling for INHBA activity between the epidermis and dermis with NPWT treatment (Figures 6A, B). Conversely, in regard to matrix remodeling, the dermis appears to be the most significant influencer of early signaling of control wounds, with significant modulation of MMP, TIMP, and BMP signaling. The subcutaneous adipose layer has the least impact during untreated control wound healing. However, this signaling is flipped upon exposure to NPWT, where there is limited additional activity within the dermis but a significant modulation of activity within the subcutaneous layer. This includes an upregulation in TGFB1 activity coming exclusively from the adipose tissue in an acute setting after NPWT treatment, whereas there is not a significant alteration in TGFB1 activity acutely in the control wounds (Figures 7A, B).

An important limitation to point out within this study is the use on only two animals, subsequently resulting in only two biological replicates (n = 2). In order to increase the rigor and external validity of the dataset, a number of steps were taken. First, experts at the Genome Core Facility processed all the tissue and extracted the RNA. A blinded bio-informaticist was utilized to process the raw data counts into DEGs. Then strict parameters were utilized to decrease the likelihood of including false positive and false negative data. Additionally, the DESeq2 package used to obtain DEGs in this study implements advance empirical Bayes methods to estimate gene-specific biological variation under minimal levels of biological replication (as low as two as stated by the authors of the package) by leveraging variance information across genes instead of treating each gene independently (Love et al., 2014; Schurch et al., 2016). Moreover, it is also important to note that the purpose was to introduce a new approach and methodology for processing and analyzing tissue, such as wound tissue, to provide more robust and accurate datasets for downstream analyses.

Although there are a number of studies to date looking into genomic expression profiles of wound healing with or without a wound interventional strategy (e.g., NPWT), there is limited information regarding a temporal and spatial perspective combined, especially in an acute setting (Derrick and Lessing, 2014; Ma et al., 2016; Brownhill et al., 2021). Moreover, there are not any standardized models for assessing wound interventions and dressings, such as that proposed in this study. There are recent advancements in genome sequencing, such as single cell sequencing or 10X spatial transcriptomics, now available that provide a wealth of information and may be more appropriate in certain scenarios. However, there are still limitations to these strategies, including lack of spatial information in single cell sequencing and a need to keep tissue/cells alive and viable, which can be difficult for large scale *in vivo* studies (Williams et al., 2022). Additionally, spatial transcriptomics can be associated with concerns of feasibility in larger studies due to the requirement of imaging every wound/tissue section, in addition to accessibility to sequencing cores with the right equipment and staff, and the economical burdens associated with these newer expensive spatial transcriptomic modalities (Williams et al., 2022). One of the many benefits of this methodology is that it can be paired with any currently available genomics core capable of performing RNA sequencing, or other genomic analysis technique preferred, such as microarrays or RT-PCR. Therefore, the methodology presented in this study provides an alternative to those unable to perform the abovementioned methods, but can also work synergistically with those methods when paired appropriately.

## 5 Conclusion

Overall, the goal of this study is to highlight the need for more targeted genomic analyses of wound healing modalities and subsequently evaluate the validity of the approach proposed. This study demonstrated a substantially different genomic perspective when stratification of wound tissue is performed rather than “Full/Bulk” analyses. Thus, there could be specific dynamic molecular mechanisms responsible for many currently utilized wound healing therapies that can be potentially identified with this methodology and tailored for future therapies. Additionally, with the increased sensitivity and specificity of a stratified tissue analysis, biomarkers for chronic and/or complex wounds could be identified and wound dressings can be tailored to release specific biomodulatory compounds within a specific region of the wound. Moreover, pairing the genomic data with additional analytical approaches such as proteomics, histology, electron microscopy, and immunolabeling permits the opportunity to not only better understand how wound interventions augment the healing process, but to also provide new insight into physiological healing at a deeper level. The results from this study and continued investigations may be able to directly translate to the clinical setting in a variety of scenarios via the optimization of therapeutic parameters for current utilized therapies, such as NPWT.

## Data Availability

Raw data uploaded to the NCBI GEO database, series ref. #GSE233863. Please use the following link to access https://www.ncbi.nlm.nih.gov/geo/query/acc.cgi?acc=GSE233863.
