# Supplementary material for: A method for temporal-spatial multivariate genomic analysis of acute wound healing via tissue stratification: a porcine negative pressure therapy pilot study
Source: Front Mol Med. 2023 Aug 31;3:1195822. doi: 10.3389/fmmed.2023.1195822 (PMC11285538; doi:10.3389/fmmed.2023.1195822)
Supplement: Supplementary file 1 [file DataSheet3.docx]

**
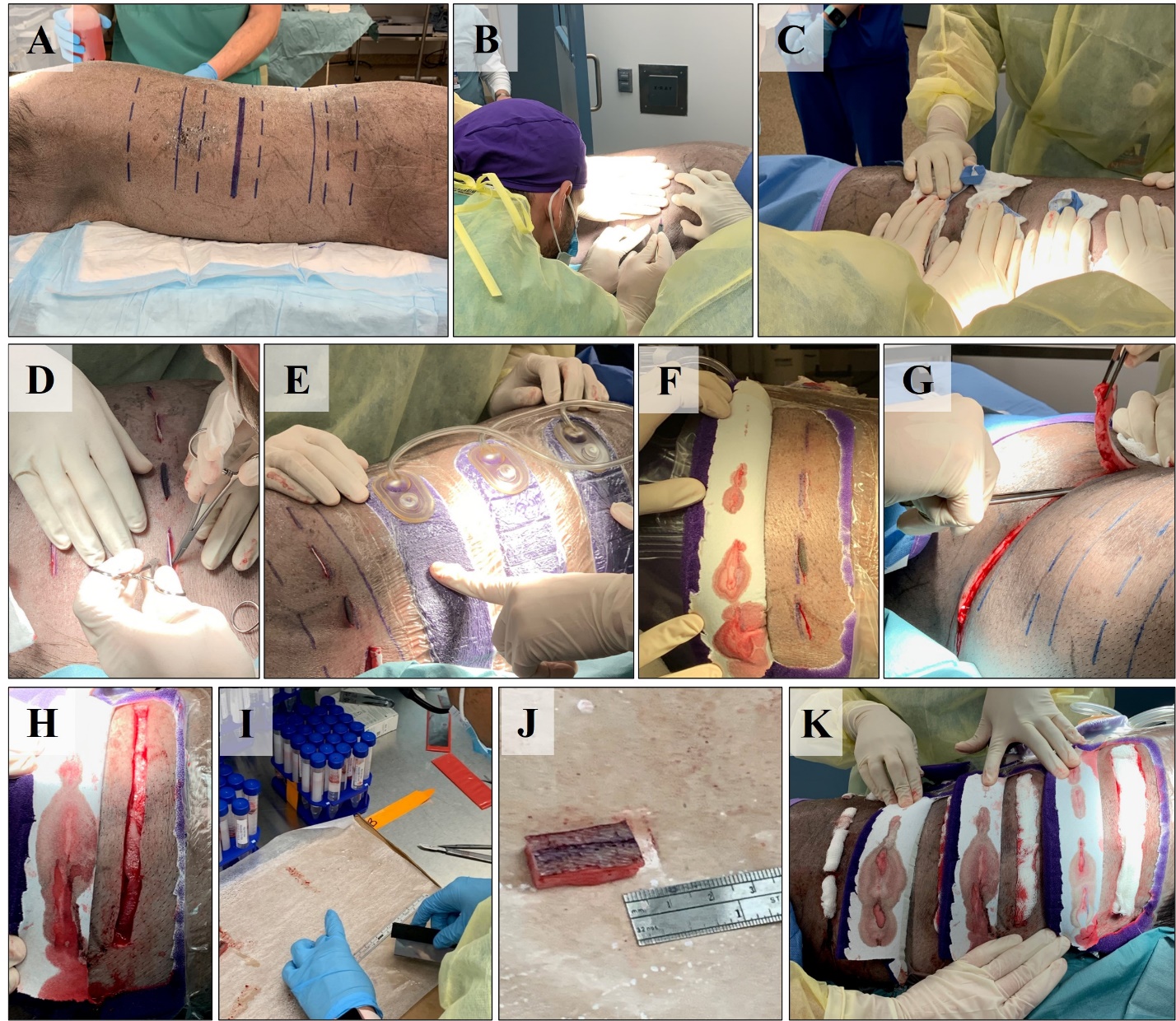
Supplemental Figure 1:** **Photographic Depiction of the Surgical Procedure Overview and Processing of Wounds.** (A) A prefabricated 3D-printed stencil was placed on the sides of the animal and the array/grid was marked with a surgical pen prior to surgery. (B) Incisional wounds were inflicted in a 4x4 array on each side of the animal with a 3D-printed scalpel guide to standardized wound depth and size. (C) Wounds were compressed for 2-minunts to control for bleeding. (D) Pre-cut wound dressing materials were inserted into each appropriate wound for each timepoint. (E) On one side of the animal, NPWT vacuum was applied for the pre-determined timepoints. (F) At each respective timepoint, vacuum was discontinued and the overlaying Prevena dressing was removed and imaged. (G) The timepoint column was removed as a single strip of tissue containing all four incisional wounds and dressing inserts at a depth of 2cm. A double-scalpel guide was used to standardize the explant width and depth, subcutaneous attachments on the deep aspect of the wounds were cut to detach wound from the animal. (H) Newly inflicted wounds from explant process were hemostatically controlled and imaged. (I) Larger strip of wound explant was processed in the surgical suite (J) via a Dermatome blade to separate the four incisional wounds into separate pieces of tissue followed by additional dissection into four smaller sections for each wound. (K) Wounds were imaged at the conclusion of the surgery to depict serous fluid removal via absorption onto the Prevena dressing.

**
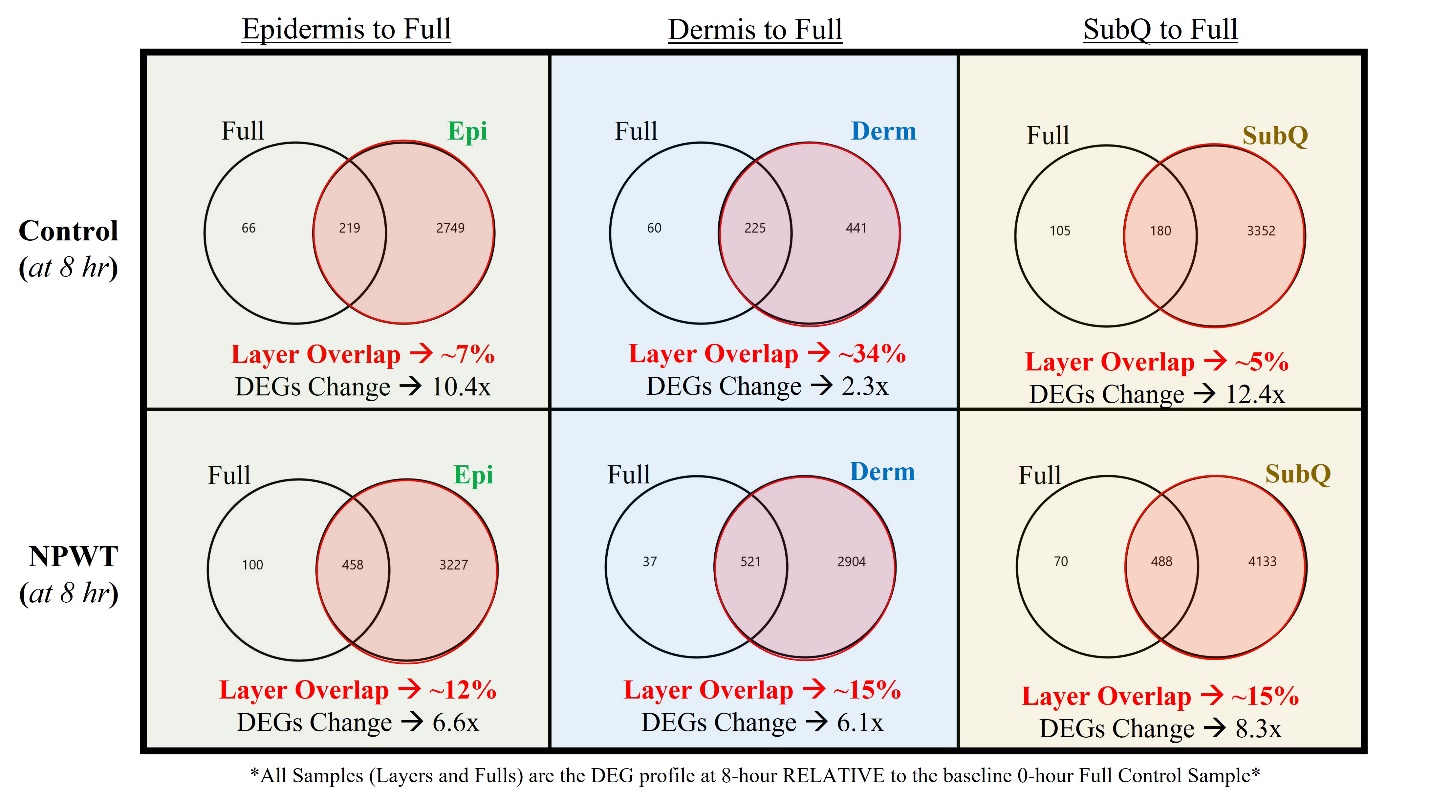
**

**Supplemental Figure 2:** **Similarity in Global Gene Expression Profiles Between Full Samples and Stratified Wound Layers.** DEG profiles from samples of either “Full” at 8-hour or Epidermis, Dermis, or SubQ at 8-hour were obtained relative to the initial baseline expressions (relative to the 0-hour baseline control sample). Then Venn Diagrams were generated for each Full and Layer pair to depict the relative overlap in DEGs for each layer pairing, including Epidermis (*Green*), Dermis (*Blue*), and SubQ (*Yellow*). Graphical depictions of DEGs are included for both the Control (*Top Row*) and NPWT-treated (*Bottom Row*) wound samples. “Layer Overlap” is the calculated percentage of common DEGs of the specified layer with the Full group (i.e., the number of similar DEGs divided by total DEGs for that specific layer). The “DEGs Changed” depicts the fold change in total number of DEGs for each layer relative to the traditional Full comparison. Of note, the graphs do not depict directionality, only overlap in DEGs that have met the predefined threshold of an FDR less than 0.05.
